# Experiential Learning to Enhance Global Health Collaboration and Student Opportunity

**DOI:** 10.5334/aogh.4537

**Published:** 2024-11-23

**Authors:** Brittney van de Water, Madelyn McLean, Colin Knutson, Rishi Srinivasan, Karl le Roux

**Affiliations:** 1 Connell School of Nursing, Schiller Institute for Integrated Science and Society, Boston College, Chestnut Hill, MA, USA; 2 Boston College, Chestnut Hill, MA, USA; 3 Primary Healthcare Directorate, University of Cape Town, Cape Town, South Africa

**Keywords:** global health, immersion, study Abroad, experiential learning

## Abstract

Students often seek opportunities to enrich their classroom learning. Providing students the chance to engage in research studies or global health projects allows for experiential enrichment. However, the impact on partners and partner sites, financial implications, and equity of student opportunity, as well as the logistical burden potentially placed on multiple parties, all need to be considered. If challenges are minimized, students can make meaningful contributions to projects, be a catalyst for partner engagement, and allow for formative learning.

## Viewpoint

### Identifying opportunities for students

Providing students opportunities outside the classroom can enhance and enrich learning. When considering global health opportunities, however, these opportunities need to consider the burden falling on the individuals and communities with which one partners. Often, such experiences can become voluntourism and a drain on already low‑resourced communities. Alternately, these experiences abroad can be formative learning opportunities for students that offer exposure to practices or experiences beyond their skill level [1]. If pitfalls can be avoided, having students support ongoing projects abroad can have significant positive effects for all parties involved.

Many undergraduate study abroad programs offer an increasing number of opportunities for students to take courses, participate in research, and provide service internationally. While there was a large reduction in students studying abroad during the coronavirus disease 2019 (COVID‑19) pandemic, program interest has almost returned to pre‑pandemic numbers. Universities on the coasts of the United States, especially the East Coast, have the highest proportion of students participating in study abroad experiences [2]. However, study abroad programs can be costly for students, depending on the university or program. The expense of semester or summer abroad courses can result in the exclusion of students from lower socioeconomic backgrounds [3]. As the interest in study abroad programs increase, it is crucial to continue expanding university support for underrepresented students to enable equitable opportunities [4].

### Setting the stage for student opportunity

For the past five years, a faculty member (B.v.d.W.) has been partnering with a rural hospital and its surrounding clinics in the Eastern Cape, South Africa, to improve tuberculosis (TB) care, specifically increasing screening, diagnosis, and uptake of TB preventive therapy. Similar to many groups around the globe, our research has found that the preventive TB care cascade is difficult to measure with current documentation systems [5–7]. Overall, our cohort of patients have had impressive TB treatment outcomes (only 12% had unsuccessful outcomes). However, individuals with TB who were coinfected with human immunodeficiency virus (HIV) have five times worse outcomes compared with individuals who were HIV negative [8]. Most recently, we implemented a household‑based screening program and used clinic‑based mobile chest x‑rays to help diagnose and rule out TB disease [9]. This led to an opportunity for students to support data collection for a month during the summer of 2022. In addition, they gained experience learning about South Africa, TB and HIV, healthcare delivery, the research process, and program and study implementation.

### Formative education and experiential learning theory

Study abroad programs often provide formative education opportunities for undergraduate students. Formative education promotes the development of students in realizing their values and purpose as whole persons [10]. This philosophical underpinning helps form students’ identities and goals. In global health research, formative education allows students to expand their knowledge of culture and its implications on community engagement [10]. Experiential learning provides an opportunity for students to learn while doing. David Kolb describes experiential learning as a four‑part “Experiential Learning Cycle” through which students learn by experiencing, reflecting, thinking, and acting. In the *experiencing* stage, students learn through experiencing what is occurring. In the *reflecting* stage, students reflect on what they have learned and attempt to understand their feelings about the experience. During the *thinking* stage, students work with the knowledge gained to make conclusions and create theories, concepts, and principles to test. Finally, through the *acting* stage, students test the theories formed and use feedback to create a new experience [10]. This model is exemplified in many study abroad programs, such as the Boston College Schiller Institute Community Engagement courses [11]. Students experience the four‑stage Experiential Learning Cycle as they learn in a classroom setting, live abroad over the summer working with community‑identified projects, and return to campus to summarize and present their findings. Throughout the course, students reflect on their experiences and think of ways to create new experiences for future students. In addition to semester‑ and summer‑long study abroad courses for credit, innovative approaches, such as research practicums, have also been developed and provide students with experiential and formative learning opportunities.

### Student voices and perspectives on south african research opportunity

Our summer trip to South Africa was a formative experience that taught us the importance of observation and reflection to understand a country and its issues. Prior to spending a month in South Africa to work on a TB study, we learned about South Africa’s history, including the legacy of Apartheid legislation and its ongoing impact, the country’s high unemployment rate of 32.9% [12], and the significant income inequality [13]. However, no amount of research or reading could have prepared us for the stark disparities and social challenges that we encountered upon arrival.

As we traveled from the airport to our accommodation in Cape Town on a multi‑lane, well‑signed highway, we drove past the townships of Khayelitsha and Langa, where many families live in dilapidated conditions. It became clear that the South Africa we had read about was reflected through this experience [14]. Seeing it with our own eyes provided a new perspective that could not be attained through readings and research. As we continued on to more affluent areas of the city, such as Camps Bay and Clifton, nestled between Table Mountain and the Atlantic Ocean, we saw the striking contrast in living conditions and demographics. The predominantly white, wealthy communities felt like a completely different world from the predominantly black townships just a few miles away [15]. Through the connections we fostered with locals, we gained a better understanding of lifestyle differences among people living in Cape Town.

In Cape Town we had the opportunity to visit the Bo‑Kaap neighborhood, a former slave quarter with a rich history. The vibrant colors of the houses, which were painted by emancipated slaves as a symbol of their freedom, stood out in stark contrast to the rest of the city. We had the chance to hear the Muslim call to prayer, the Adhan, over the intercom system connecting the entire community, which was a moving experience and different from suburban Boston or Minneapolis. We also had the opportunity to visit Victoria Hospital, a government‑run facility. We shadowed doctors on the pediatric unit and learned about common illnesses that are not as common in the United States, such as TB and HIV. The layout of the hospital differed from the typical US hospital with hospital beds in an open ward instead of individualized rooms. The only patients put in small independent rooms were patients who were immunocompromised or contagious.

### Eastern cape

The highlight of the trip was the visit to Zithulele District Hospital, a rural village in Eastern Cape, South Africa. The journey from Durban, which involved navigating bumpy roads and dodging potholes for eight hours, was worth it for the opportunity to work one‑on‑one with Dr. Karl le Roux and Dr. Lbé van Niekerk. We learned about – and experienced – patient care and clinical research in the context of rural medicine. It was enlightening to see how medical care and public health were intertwined to serve this rural population. Watching the doctors and community health workers treat every patient with respect and truly connect with their culture and beliefs inspired us and shaped the kind of care providers we want to become.

Local community health workers (CHWs) who spoke the native isiXhosa language were incorporated into the health system. This was essential for effective interaction with patients and families, and for providing proper resources to educate the community about TB and HIV. By having these CHWs, the team was able to establish trust and educate patients in a relatable manner. By the end of our time in the former Transkei, we learned to introduce ourselves in isiXhosa and how Xhosa customs are incorporated into rural medicine. For example, people may consult traditional healers (sangomas) prior to seeking modern medical care, often resulting in treatment delays [16]. Supporting this research study challenged our assumptions on the feasibility and efficacy of community interventions. We witnessed the importance of cultural sensitivity and community engagement in measuring the success of public health initiatives. This experience led us to question the traditional top‑down approach to healthcare provision and consider the potential of a community‑led approach. For example, we observed how the involvement of local health workers significantly improved participation rates and trust in the project, which has significant implications for future global health programs.

Beyond the work at the hospital, we also had the opportunity to immerse ourselves in the Eastern Cape community, visiting caves, coves, and rock formations, and enjoying activities with the Zithulele Hospital health team. Despite the challenges with internet access due to “load shedding” (planned electrical outages due to limited generating capacity in the country), we appreciated the opportunity to disconnect from the digital world. Above all, it was the connections we fostered that made the experience rewarding. We were invited by members of the Zithulele Hospital community to watch the Cape Town Stormers rugby team play in the final on TV. The same group later invited us to their Bible Study and gave us the opportunity to play in their frisbee tournament – all welcoming experiences.

### Student final thoughts

The best part about our experience was the constant exposure to different aspects of healthcare within South Africa. We were able to compare and contrast the forms of healthcare needed and administered in this region, and it was helpful to consider possibilities beyond our known norms when thinking about our future and when we ultimately enter the workforce. Overall, our trip to South Africa was empowering. Connecting with South Africans one‑on‑one and immersing ourselves in their day‑to‑day lives allowed us to gain knowledge in deeper ways than many of our courses at home have allowed us. Following this experience, both of us are inspired to create change, particularly in addressing health disparities, more than ever. However, we have also learned about the obstacles to change – whether it be financial, cultural, or political – and are beginning to understand the considerable thought and effort required to implement reform. Our trip taught us through individuals, such as Dr. Karl, Dr. Lbe, and the community health workers, that this change can become reality.

This research study focused on promoting community and public health, yet it was a formative education experience allowing students to develop and realize their values and purpose as whole persons. This experiential learning opportunity helped to form students’ identities and goals through utilizing cultural resources [10]. In global health research, this allows students to learn how to understand their own culture and that of other communities.

### The experience of the rural south african researchers and clinicians of hosting students

For researchers and clinicians working in rural and less resourced contexts, the arrival of students can be helpful and motivating. Yet equally, students can at times become a burden and a significant drain on time, scarce resources, and morale. It is critical that students understand that any exchange opportunity is a privilege. Setting up an exchange requires significant thought, time, and input by receiving researchers and clinicians, who are usually already stretched and overworked. Although students usually intend to be useful and believe that they come to help, it usually takes several weeks – or sometimes months – for them to find their feet and for their assistance to be genuinely useful to the receiving clinicians/researchers and community. Students leaving for exchanges and electives in under‑resourced parts of the world should reflect on Table 1, where we list characteristics and actions that make students either a burden or helpful.

**Table 1:** Characteristics and interactions of burdensome versus effortless students in low resource settings – from the perspective of hosting clinicians/researchers.

	STUDENTS WHO ARE A BURDEN	STUDENTS WHO ARE HELPFUL
Time spent on an abroad elective	A month or less	At least 6–8 weeks
Before elective – communication and email correspondence	Sending many emails with a myriad of questions, which have already been answered in information sheets or on elective websites Being demanding and pushy Expressing frustration when questions or emails are not answered immediately	Politely persistent, understanding that receiving clinicians/researchers are busy and are not always on their email Sending only one or two emails asking important practical questions, and following up with a gentle reminder if not answered
Student characteristics	Judgmental, arrogant, demanding, critical Appear to believe they have the answers, trying to show off how smart they are	Humble, thoughtful, considerate Attitude of enquiry, wanting to learn and understand, asking thoughtful questions Prepared to give/receive constructive criticism when requested
Work and interactions	Focused on their own goals and experience/research priorities Not prepared to do anything beyond what they believe is their “job description” Requiring constant guidance and “hand‑holding” Appearing to prefer digital interaction with distant friends to living in the present	Prepared to identify needs and help out with anything that is needed – from entering data to washing coffee cups Able to work on their own, take initiative, but also knowing when to ask for help Immersing themselves in the local community and culture – participating in local events and showing a genuine interest in all people

Global education requires interdisciplinary training and reflection. The commitment to learning before, during, and after the experience abroad is crucial [17]. Students have the opportunity for holistic learning through preparing before the trip, actively learning while abroad, and reflecting on the experience afterward. This commitment enables a mutually beneficial relationship with the local communities the study abroad students work with, particularly in global health education and research.

In summary, humble, thoughtful students whose attitude is one of wanting to learn, who follow researchers’ guidance – yet can work independently – and who are able to identify needs and help in a broad variety of ways to support projects are a pleasure to host.

## Conclusion

The rich experiences students – and all individuals involved – gain from time immersed in another country, culture, and simply away from the daily routines of life, can be rewarding. Having time to critically evaluate one’s own life experiences and choices, reasons for choosing a certain major or career path, and having time to ponder questions in a vastly different landscape to what is known can provide significant growth opportunity. Experiential learning opportunities abroad also create the potential for additional collaborations and strengthen existing partnerships. For example, although the faculty member was unable to travel while these students were abroad, it led to heightened communication between colleagues and additional support for the ongoing study. This perspective builds upon existing literature on experiential learning in global health by examining the student experience and integrating it with faculty and community partner perspectives. It highlights the importance of community engagement and reflection in creating impactful learning experiences for students and positive engagements for all.

Using Kolb’s experiential learning cycle [18] and this unique opportunity where students engaged in an existing research study, students had the opportunity to reflect on their predeparture readings. Then, students were able to act and critically engage on the implementation of a TB study in a high burden setting. Finally, students had the time and opportunity to work with the knowledge gained to develop conclusions about their experiences. Hopefully, they will take this formative experience forward with them throughout their personal and professional experiences.

The importance of one‑on‑one relationships cannot be underscored enough, in global health research, in healthcare, and in student mentorship. However, systematic support for projects and the development of student travel grants must be built into university programs and study budgets. This will ensure that immersion programs, research opportunities, and study‑abroad programs are available, equitable, and sustainable. These opportunities can be a quadruple win for students, faculty, universities, and communities.
